# Graph4Med: a web application and a graph database for visualizing and analyzing medical databases

**DOI:** 10.1186/s12859-022-05092-0

**Published:** 2022-12-12

**Authors:** Jero Schäfer, Ming Tang, Danny Luu, Anke Katharina Bergmann, Lena Wiese

**Affiliations:** 1 Institute of Computer Science, Goethe-Universität Frankfurt, Frankfurt am Main, Germany; 2 Department of Human Genetics, Hannover Medical School, Hannover, Germany; 3 L3S Research Center, Leibniz Universität Hannover, Hannover, Germany; 4 Bioinformatics Group, Fraunhofer ITEM, Hannover, Germany

**Keywords:** Graph database, Medical database, Data exploration, Visualization, Web application

## Abstract

**Background:**

Medical databases normally contain large amounts of data in a variety of forms. Although they grant significant insights into diagnosis and treatment, implementing data exploration into current medical databases is challenging since these are often based on a relational schema and cannot be used to easily extract information for cohort analysis and visualization. As a consequence, valuable information regarding cohort distribution or patient similarity may be missed. With the rapid advancement of biomedical technologies, new forms of data from methods such as Next Generation Sequencing (NGS) or chromosome microarray (array CGH) are constantly being generated; hence it can be expected that the amount and complexity of medical data will rise and bring relational database systems to a limit.

**Description:**

We present Graph4Med, a web application that relies on a graph database obtained by transforming a relational database. Graph4Med provides a straightforward visualization and analysis of a selected patient cohort. Our use case is a database of pediatric Acute Lymphoblastic Leukemia (ALL). Along routine patients’ health records it also contains results of latest technologies such as NGS data. We developed a suitable graph data schema to convert the relational data into a graph data structure and store it in Neo4j. We used NeoDash to build a dashboard for querying and displaying patients’ cohort analysis. This way our tool (1) quickly displays the overview of patients’ cohort information such as distributions of gender, age, mutations (fusions), diagnosis; (2) provides mutation (fusion) based similarity search and display in a maneuverable graph; (3) generates an interactive graph of any selected patient and facilitates the identification of interesting patterns among patients.

**Conclusion:**

We demonstrate the feasibility and advantages of a graph database for storing and querying medical databases. Our dashboard allows a fast and interactive analysis and visualization of complex medical data. It is especially useful for patients similarity search based on mutations (fusions), of which vast amounts of data have been generated by NGS in recent years. It can discover relationships and patterns in patients cohorts that are normally hard to grasp. Expanding Graph4Med to more medical databases will bring novel insights into diagnostic and research.

## Background

Medical databases are not only vitally important for providing accurate and timely health services but also crucial for an improvement of the work flow for doctors, researchers and health care providers. Managing a health database system is challenging because it needs to ensure (1) real-time access and analysis, (2) data security and sharing, (3) patient privacy while having to deal with very different data formats and users [1]. Traditional medical databases are usually relational or network-based. They are designed to manage the information that stores different data regarding a single entity. However as the volume and diversity of medical data continue to expand exponentially, people realize that a relational model actually keeps “healthcare data locked, isolated and unused” [2]. More and more healthcare providers are migrating from relational to non-relational database systems like graph databases and document data stores [3–5].

### Medical graph databases

With the increasing amount of heterogeneous biological data obtained by novel technologies in the medical sector, graph databases have gained more attention as flexible and feasible storage systems [6] that help to find and understand complex hidden relationships [7]. Biological pathways can also be modeled more efficiently in a graph database than in a traditional relational database, which results in an increased query performance when traversing the knowledge graph [8]. The integration of multi-omics data provides the ability to extract new knowledge from data but is challenging due to the high diversity and complexity of such data and requires novel approaches (e.g., as provided by graph database systems) [9]. One of the most popular graph database systems is the Neo4j graph data platform [10] (cf. Graph Databases). It was chosen for realizing medical data applications around the management of biological knowledge bases [8, 11] or the integration of data from multiple sources [12].

The usage of a graph database such as Neo4j to store, manage and query medical data often serves the purpose of building a backbone for a web application with easy user access. A web-based dashboard is a powerful tool for visualizing and analyzing the graph data as it makes the stored data available to the users in a comprehensible fashion by abstracting from the underlying graph database technology. Bukhari et al. [12] have used a Neo4j database to implement such an intuitive dashboard on top of it for browsing and visualizing immunological data in plots supporting the automatic translation of natural language queries to Cypher queries.Their web application lets users view immunology-related data, e.g., age distributions of subjects in a study, via a graph-based and natural language query interface for a more intuitive usage. Our approach, in contrast, uses a dashboard that retrieves data for rendering with Cypher queries, that the user does not need to interact with but can if they are familiar with Cypher, for multiple visual representations of data in one page. The purpose of LinkedImm is to integrate different data sources into a linked graph, whereas our tool focuses on the analysis of a cohort of patients obtained from a relational database and transformed into a graph model.

The graph database BioGraphDB [13–15] also relies on Neo4j as one of the core technologies and has been used to build the BioGraph [16] web application, which allows users to interactively query and analyze the integrated biological data, e.g., microRNA or protein sequence data. Similar to LinkedImm, the BioGraph integrates data from multiple sources into a data graph with a manually derived graph schema to model the relationships between biological entities like genes and proteins. The schema was developed according to the results of the ETL processes whereas our proposal gives a generally applicable methodology to transform a relational to a graph data schema. The web application on top of BioGraph offers also only single visualizations of data at a time, that can be retrieved with several predefined queries with parameters or freely entered queries. Our dashboard is capable of visualizing an aggregation of different aspects for a more comfortable work flow. Another platform displaying multi-omics data in a web application has been developed in Graphomics  [11]. It combines a Neo4j database that maps the multi-omics data to a graph of connected entities, reactions and pathways with a relational SQLite database that stores the final results.

### Medical background

The integration of Next Generation Sequencing (NGS) and related technologies to medicine have revolutionized the field and made personalized medicine possible. As large amounts of data are being generated and added to medical databases, their analysis and visualization becomes increasingly challenging. This greatly hampers our efforts to take full advantage of these new technologies. A specific example would be the pediatric acute lymphoblastic leukemia (ALL) database that we used in this study. In acute lymphoblastic leukemia the most common genetic drivers are gene fusions while mutations and copy number variations may also contribute [17]. Over the last years, diagnostic samples of ALL were analyzed by RNA sequencing, panel sequencing and/or arrayCGH. RNA sequencing detects gene fusions much more efficiently than the traditional methods such as karyotyping or Fluorescence in situ hybridization (FISH). Panel sequencing identifies mutations in selected DNA regions of interests. ArrayCGH is a powerful method that detects losses or gains of genomic regions. All these new technologies play critical roles in providing diagnostic, prognostic and treatment information. For example, fusion result is one of the main criteria to stratify leukemia patients and identify patient similarities [17]. However, the current relational databases lack the capacity to search and analyze patients based on fusion/mutation types. In contrast, similarity searches are simplified by using a graph-based database structure.

### Our contribution

We introduce Graph4Med, a user-friendly, graph-based visualization tool for the analysis of a cohort of patients. In particular, we extracted pediatric ALL cases from a relational database and transformed them into a graph schema tailored to our use case, which was derived from the relational schema. The extracted cohort was then stored in a Neo4j graph database and a web-based dashboard was built with NeoDash, a Neo4j dashboard building tool [18]. The rest of this work is organized as follows: First, we outline the limitations of the current relational system and the benefits of using a graph database in our use case in section “Use case scenario”. Then the section “Implementation” describes the process of modeling the graph database schema from the transformation of the relational schema into a graph schema, which is further converted into the final graph model. Then in the “Results” section the system architecture and the built application dashboard are elucidated. We further comment on the usability, significance and limitations of our implementation in the “Discussion”. Finally, a summary of our contribution is given in the “Conclusion”.

## Use case scenario

### Current relational system

Currently our medical partner uses a system that employs a relational database and a graphical interface to interact with the patient data. According to the relational schema, the user can browse different concepts and navigate through the case of an individual patient by viewing data about the diagnoses, samples, tests, analyses or prescriptions in the form of tabular or unstructured data. Although it is possible to alter the stored records, there are no straightforward visualizations other than tables or sheets to obtain a comprehensive overview. This leads to overly complex and time intense work for the user to identify key aspects for the diagnosis or treatment of the subject. To this end, a dashboard improves the work flow by displaying the required and most valuable information concisely and user-friendly.

In particular, the desired information in many scenarios is often scattered across multiple tables due to database normalization. The information inside these tables are only shown separately in the interface. To address certain questions, clinicians and researchers have to acquire information from different tables. However, the combination of multiple tables via joins in queries, that should aid to directly derive the same solution, often also impedes the work flow as these tables are quite overloaded and, thus, inefficient to work with. Additionally, the resulting joined table might contain redundant information inflating the amount of information the user has to deal with. Especially, if querying for a subgroup of patients, the non-redundant information usually needs to be aggregated carefully as otherwise records with redundant information are returned. To overcome these issues, the proposed system provides access to the information in an implicitly non-redundant fashion by using a graph database.

Further use cases not addressed by the former system are to examine common features or correlations inside subgroups of patients and not just the individuals, which is an important aspect for research in general. Particularly regarding ALL, we believe that it is crucial to investigate on common fusions or mutations to gain valuable insights for diagnostics and treatment opportunities. Hence, the system is required to support the search for a specific subgroup by fusions and appropriate display of the gathered information, for instance, by applying additional filters based on the age at diagnosis, sex or aneuploidy of the patients. If the case of an individual patient is identified to be of high interest, the next step usually is to find other cases being similar to the target patient. This functionality is also not in the scope of the old relational-based system as it would require a set of complex SQL queries or a separate application program to measure similarity between patients.

### Graph databases

Graph databases manage data by employing a graph data model using graph structures for the logical representation of data and their schema [19]. The essential part comes from the mathematical formulation of graph theory that supplies the abstract data type. Formally, a graph consists of a set of nodes (or vertices) and a set of edges to connect pairs of nodes. Edges can have a direction pointing from one node to another or be undirected. In a labeled-property graph, both the nodes and edges are labeled and can have additional properties in the form of key-value pairs further characterizing the entities (nodes) and relationships (edges). In the context of a database, data are then obtained by formulating queries against the graph and data manipulations are performed by graph transformation operations.

One of the benefits of using a graph data model is that related information can be queried more easily. Starting from an individual patient or a common concept, e.g., the detection of a certain fusion or mutation, the graph can be traversed along multiple relationships step-by-step without having to consider unrelated data. The index-free adjacency ensures that the neighbors of a node (i.e., connected via an edge) can be directly accessed from the node itself and, thus, the lookup performance does not depend on the size of the graph resulting in a better scalability. Furthermore, queries are more flexible regarding the grouping and aggregation of data by returning subgraphs as query results. This capability implicitly eliminates duplicate answers on-the-fly as the same node is just returned once but with possibly multiple relationships/paths to other nodes. For example, consider a scenario of multiple chained one-to-many relationships, e.g., various analyses, that themselves might have multiple results, can be done for each patient. In relational databases the grouping for findings per patient and analysis would lead to a table with potentially redundant information as each patient occurs once for each analysis. The graph database, in contrast, would return a subgraph of patient nodes with paths via analysis nodes to result nodes. The graph structure inherently yields a powerful possibility for visualization of such subgraphs that facilitates the identification of complex relationships in the data by the user.

Neo4j is our choice of graph database management system that “stores and manages data in its more natural, connected state, maintaining data relationships that deliver lightning-fast queries, deeper context for analytics, and a pain-free modifiable data model” [10]. Neo4j is available in an open-source version and comes with a native graph database, the graph query language Cypher and libraries for graph analytics. It has a vivid community, a broad variety of tools and extensions, and is one of the most popular systems for storing data with a graph data model. The corresponding query language comes with an intuitive and logical syntax that is easy to learn and understand (e.g., documentation at https://Neo4j.com/docs/). This is important as our dashboard will use Cypher queries to populate the reports with data directly obtained from the database.

## Implementation

### Relational database schema

**Fig. 1:**
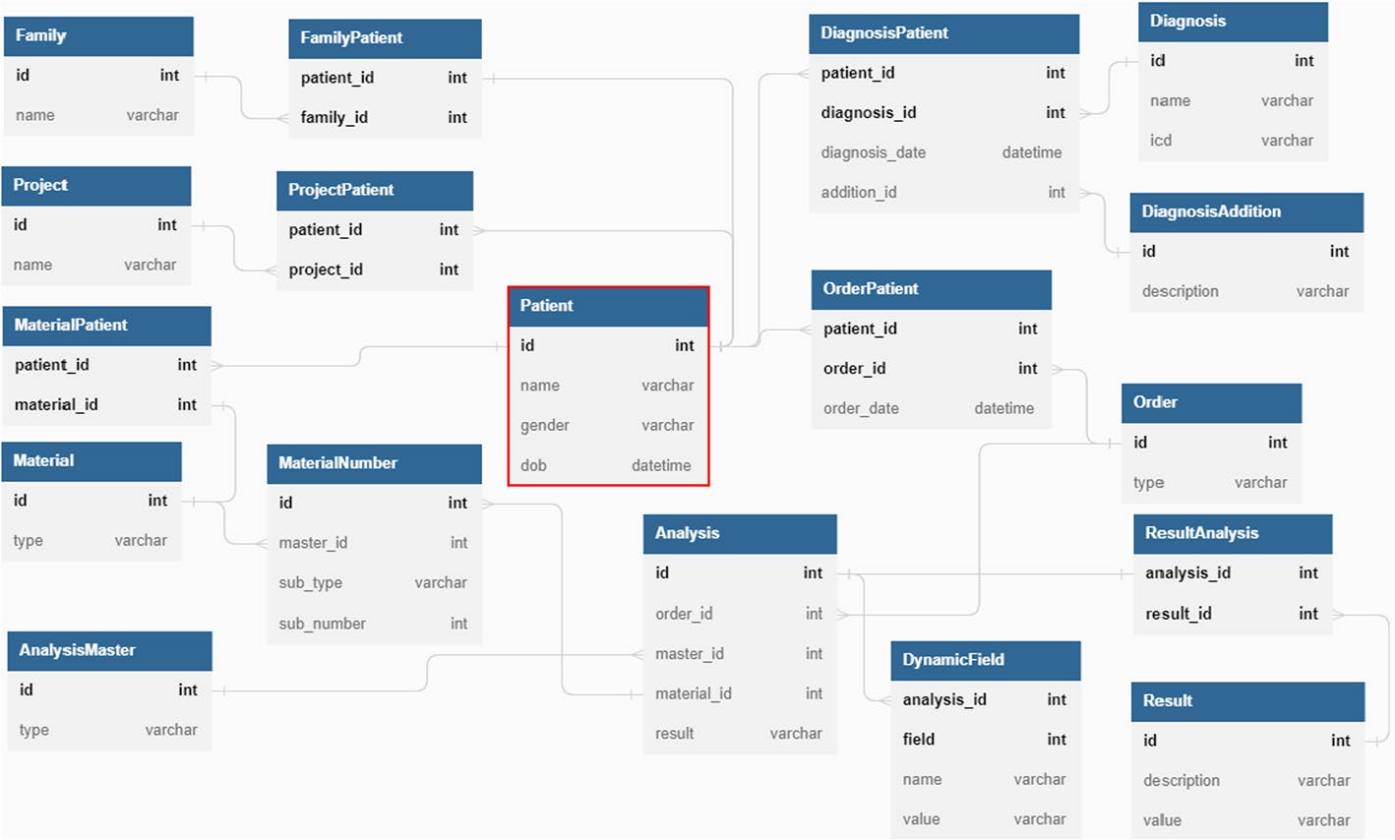
Relational database schema. The schema of the relational database with primary key (PK) attributes indicated by bold names and foreign key (FK) relationships indicated by the grey lines connecting FK and PK attributes

The data underlying the dashboard application was extracted from a relational database and mapped into a suitable graph data model. Figure 1 shows a simplified version of the relational database schema that was restricted to only comprise the features of the entities and core relationships between the different entities present that are relevant for the visualization of the ALL cases in the dashboard. Formally, the relational database schema $\textbf{R}$ consists of the following main relations: Patient(id, name, gender, dob), Project(id, name), Family(id, name), Order(id, type), Diagnosis(id, name, icd), DiagnosisAddition(id, description), AnalysisMaster(id, type), Analysis(id, order_id, master_id, material_id, result), DynamicField(analysis_id, field, name, value), Material(id, type), MaterialNumber(id, master_id, sub_type, sub_number) and Result(id, description, value).

These relations model the logical entities of our use case and the other relations are associative relations that implement the logical one-to-many or many-to-many relationships. Such relationships occur for the membership of patients in families and projects, the assignment of the patients’ diagnoses incl. addition, orders and materials, or the relation of analyses to results, that may aggregate multiple analytical results. The relation that represents the patient entity plays a central role (marked red) in the designed relational model. The other relations modeling logical entities can be differentiated into patient-specific or patient-agnostic relations. The relations Project, Family, Diagnosis, DiagnosisAddition and AnalysisMaster are patient-agnostic as they (generally) do not store information that depends on a specific patient but potentially link to every patient. The tuple (12345, ’Leukemia Research’) in the relation Project, for instance, represents a research project named ’Leukemia Research’ with id 12345 and multiple patients as subjects. In contrast to this, the relations Order, Analysis, Result, DynamicField, Material and MaterialNumber are patient-specific and store information that belong to a specific patient. For instance, the relation Material can contain a tuple (98765, ’DNA’) representing a DNA sample with id 98765 that was obtained from a patient (as mapped by the MaterialPatient relation).

### Schema graph transformation

Similar to Definition 1 in [20], we constructed a relational schema graph $\textbf{RG} = \langle N, E\rangle$ for the relational database schema $\textbf{R}$ with a node $n_a$ for each of the attributes $a \in X_i$ of each relation schema $R_i(X_i)$ in $\textbf{R}$. Each node $n_a$ is labeled with the name of the relation $R_i$ followed by a dot and the name of the attribute *a*, i.e. $R_i.a$. Before introducing edges, we merge the nodes of composite PK attributes into single nodes which are then labeled with $R_i.PK$. This reduced the amount of nodes and edges for a simpler representation of the schema. Furthermore, the transformation of the schema graph to the graph data schema is simplified by the merge of PK attributes when creating the nodes representing the different entities. In the following, we refer to both single and composite PK attribute nodes as PK nodes. Also, we did not consider composite FKs but they could be handled in the same way as composite PKs by merging them into one single FK node. The general transformation steps towards the final graph data schema are:

1. Create nodes labeled with $R_i.a$ for relations $R_i$ and their attributes *a*.
2. Merge nodes of composite PK attributes into single PK nodes.
3. Create directed edges from PK nodes to nodes of other attributes *a* of the same relation $R_i$.
4. Create directed edges from FK nodes to the respective PK nodes.
5. Merge sinks (i.e. nodes without any outgoing edges) that are all connected to the same PK node and have only one incoming edge into one node labeled $R_i.attributes$.
6. Merge PK hubs (i.e. nodes with incoming and outgoing edges) labeled $R_i.a$ with only one outgoing edge to a (merged) sink with these sinks. The new entity nodes are labeled $R_i$ and contain all attributes from the previously merged hub and sink.
7. Replace sources *n* (i.e. nodes without incoming edges) that are connected to exactly two entity nodes *p* and *q* and these two edges by an undirected edge *e* connecting *p* and *q* directly.
  Case 1: If *n* has no other edges, no other actions are required.
  Case 2: If *n* has an edge to a (merged) sink *m*, add the attribute(s) represented by *m* as property to *e* and remove *m*, too.
  Case 3: If *n* has an edge to a hub *m* with only one other edge to an entity node *r* containing only one additional attribute *a* next to the identifying attribute(s), add *a* as property to *e* and remove *m* and *r* from *N*.
  If none of the above cases is applicable, no merge is performed.
8. Resolve FK relations by edges:
  Case 1: Replace FK relations indicated by hubs $n_h$ with one incoming edge from an entity node *m* and one outgoing edge to an entity node *o* by an undirected edge directly connecting *m* and *o*.
  Case 2: If the FK relations is a source $n_s$ labeled $R_i.a$ with outgoing edges to an entity node *m* and to a (merged) sink *o*, first merge $n_s$ and *o* (with all attributes except the FK attribute) into an entity node $R_i$. Then, connect the entity nodes *m* and $R_i$ with an undirected edge.

**Fig. 2:**
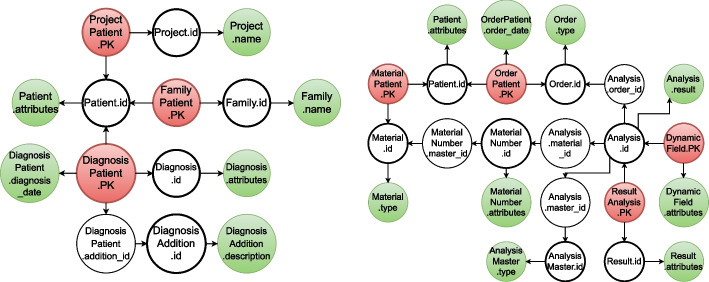
Schema graph. The constructed and compressed schema graph $\textbf{RG}$ which is split into two parts $\mathbf {RG_1} \cup \mathbf {RG_2}$ (left and right, respectively). Nodes with a thick outline represent PK attributes. Sinks are colored green and sources are colored red

The obtained schema graph $\textbf{RG}$ after applying steps 1-5 is shown in Fig. 2 but was split into two parts $\mathbf {RG_1} \cup \mathbf {RG_2}$ for a less overloaded visualization. The left part of the figure displays the schema graph around the Patient, Family, Project, Diagnosis and DiagnosisAddition relations and the right part displays the schema graph restricted to the Patient and the other relations. The PK property of attributes represented by the nodes are highlighted by a thicker outline (e.g., the node labeled *Patient*.*id*). The direct edges of $\textbf{RG}$ visualized by arrows between the nodes indicate either the FK relationships between attributes or the relationship of PK to non-key attributes (e.g., the node *OrderPatient*.*PK* comprises the two attributes OrderPatient.patient_id and OrderPatient.order_id), which are references to patients and orders, respectively. It also links the PK to the OrderPatient.order_date attribute characterizing the date of order request. The (merged) sinks are colored green and the source are colored red.

**Fig. 3:**
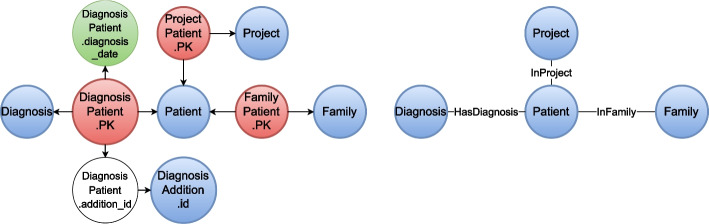
Schema graph $\mathbf {RG_1}$ transformation. The transformation of the schema graph $\mathbf {RG_1}$ is depicted in two steps. The left panel shows how hubs and directly connected sinks were merged into new entity nodes (blue) in $\mathbf {RG_1}$ according to transformation step 6 (e.g., nodes *Patient*.*id* and *Patient*.*attributes* were merged into node *Patient*). Sources were transformed into edges connecting the previously created nodes directly (e.g., *ProjectPatient*.*PK* became the edge labeled *InProject*) as shown in the right panel (step 7)

Figure 3 shows the application of the transformation steps 6 and 7 to the first part $\mathbf {RG_1}$ of the schema graph (left graph in Fig. 2) from left (step 6) to right (step 7). At the first stage, the nodes representing the patient, project, family, diagnosis and diagnosis addition entities were created by merging the hubs, that are PK nodes, with the connected attributes. This aggregated the entity-specific properties in one node. The source nodes between exactly two of the new entity nodes (blue colored nodes in Fig. 3) were resolved as edges modeling the relationship between objects of the two entities. The simplest scenario (step 7.1) occured for transforming the sources labeled *ProjectPatient*.*PK* and *FamilyPatient*.*PK* into the relationships *InProject* and *InFamily* between patients and projects/families stating that a patient was participating in a research project or was member of a family, respectively.

For the third source labeled *DiagnosisPatient*.*PK* the transformation steps 7.2 and 7.3 were applicable. In absence of the DiagnosisAddition relation, the direct relationship between the *Patient* and *Diagnosis* node would simply be established by transforming the source according to step 7.2. The relationship *HasDiagnosis* would then also incorporate the additional property for the date at which the certain diagnosis was made for the specific patient. Considering also the diagnosis addition, the ternary relationship could not be modeled by a single relationship between two of the affected entities if the DiagnosisAddition relation stored more than just an id and description (i.e. further attributes regarding the characteristics of the addition or FK attributes linking to other relations). However, the attributes DiagnosisPatient.addition and DiagnosisAddition.id constituting the FK relationship between a patient’s diagnosis and the (optional) addition to the diagnosis were consumed as defined in step 7.3 because the DiagnosisAddition.description was pulled into *HasDiagnosis* as an additional attribute of the relationship, too.

**Fig. 4:**
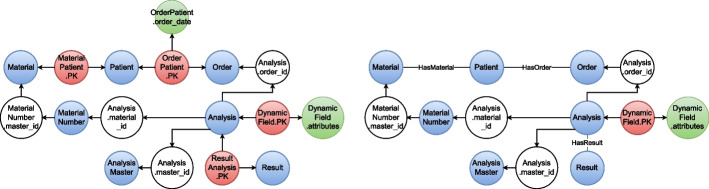
Schema graph $\mathbf {RG_2}$ transformation. The transformation of the schema graph $\mathbf {RG_2}$ is depicted in two steps. The left panel displays the merge of hubs and directly connected sinks into new entity nodes (blue) in $\mathbf {RG_2}$ according to transformation step 6 (e.g., nodes *Patient*.*id* and *Patient*.*attributes* were merged into node *Patient*). Sources were transformed into edges connecting the previously created nodes directly (e.g., *OrderPatient*.*PK* and $OrderPatient.order\_date$ became the edge labeled *HasOrder* with attribute $order\_date$) as shown in the right panel (step 7)

The same transformations were applied to the other half of $\textbf{RG}$. This is visualized by the two graphs in Fig. 4 after subsequent application of each of the two previously described transformation steps 6 and 7. From the transformation step 6, we obtained the nodes *Order*, *Analysis*, *Result*, *AnalysisMaster*, *MaterialNumber*, *Material* and of course *Patient* (which was already shown in Fig. 3) with the respective attributes. Following the rules for transformation step 7, the sources *OrderPatient*.*PK*, *ResultAnalysis*.*PK* and *MaterialPatient*.*PK* were converted into edges labeled *HasOrder*, *HasResult* and *HasMaterial*, respectively.

**Fig. 5:**
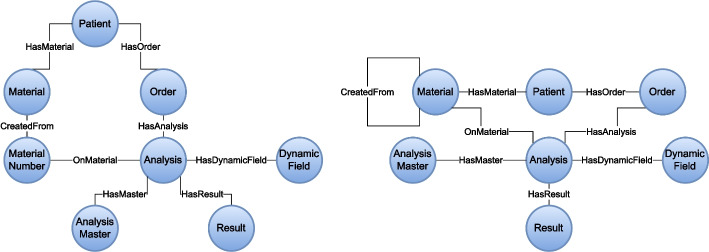
Schema graph $\mathbf {RG_2}$ transformation. The further transformation of the schema graph $\mathbf {RG_2}$ is depicted in two steps. The left panel depicts how the remaining FK relations between some entities were resolved and how the respective nodes were replaced by relationships between the entities in $\mathbf {RG_2}$ according to transformation step 8. For instance, the reference from *Analysis* to *Order* via the FK $Analysis.order\_id$ was transformed to *HasAnalysis*. The graph data model was further refined and simplified by structural changes (e.g., pulling the *AnalysisMaster* into the *Analysis* entity) as depicted in the right panel

Figure 5 visualizes the further processing of $\mathbf {RG_2}$ by the previously defined conversion of FK relations into edges (step 8). In particular, the four hubs labeled $MaterialNumber.master\_id$, $Analysis.material\_id$, $Analysis.master\_id$ and $Analysis.order\_id$ fulfill the condition of linking two entities (i.e. they represent a FK relation between them), and, thus, were resolved as relationships in the graph data model according to step 8.1. These new edges, that were labeled *CreatedFrom*, *OnMaterial*, *HasMaster* and *HasAnalysis*, replace the four hub nodes in $\mathbf {RG_2}$ depicted in the left-hand panel of Fig. 5. Furthermore, based on the source *DynamicField*.*PK*, that covers the analysis_id and field attribute of the DynamicField relation, and the sink *DynamicField*.*attributes* a new entity *DynamicField* and a new relationship *HasDynamicField* going out from it towards the entity *Analysis* were added to $\mathbf {RG_2}$ (step 8.2).

### Use case specific data model adaptation

Under the consideration that the AnalysisMaster relation only contained a single attribute description next to the id attribute with the sole purpose of identifying each record uniquely, the same information from the corresponding *AnalysisMaster* entity could also be incorporated into each *Analysis* entity directly by declaring the description about a certain type of analysis as an attribute of the *Analysis* entity logically replacing the *AnalysisMaster* entity. As alternative to this strategy, we specified a node label for each of the different types of analysis that all inherit attributes and relationships from the general *Analysis* node label for a more fine-grained and intuitive modeling. On the one hand, this allowed the formulation of Cypher queries that either traverse analysis nodes regardless of the specific type of analysis or traverse nodes of a specific analysis type only. The definition of the data model in this way, on the other hand, could also utilize even more sophisticated, deeper taxonomies. The effect of this restructuring is also shown in the right panel of Fig. 5 where the *AnalysisMaster* entity was “absorbed” by the *Analysis* entity that now also represented the node labels of the different analysis types (not visualized in this figure for the sake of simplicity).

Another transformation was applied to the entity nodes representing the material hierarchy obtained as biological samples from a patient (e.g., DNA or RNA material, from which sub-materials are produced by cultivation or preparation). We did not further distinguish between the subtypes of materials such as preparation or cultivation but rather model them as material themselves which were obtained from a main material. Hence, the *MaterialNumber* entity was merged into the *Material* entity and then represented main materials and sub-materials produced from main materials. A node in the Neo4j database with the label *Material* is then either a main material with a unique number as *id* and a *type* value or it is a sub-material with an additional *subnumber* attribute. The optionality of such properties as well as the introduced self-reference (i.e. the edge labeled *CreatedFrom* in the graph on the right panel in Fig. 5) appropriately mirrored the native dependence in the material hierarchy. Here, we decided not to model the materials analogously to the analyses with additional labels for the different types of material. This avoids an inflation of the model by too many node labels for the more than 50 material types.

To model the peculiarities of the different types of analyses recorded in the medical database which manifest in diverse results (e.g., mutations indicated by potentially multiple fusions as detected in the context of an RNA sequence analysis or the determined karyotype information based on an Array-CGH analysis) the relational schema contains multiple relations storing that analysis-dependent information as key-value pairs. For the implementation of our system, we, first, aggregated them logically into one placeholder relation called DynamicField representing all the different relations with specific key-value pairs for the results of the analyses. This relation resulted in the applied transformation of the relational schema to the graph data model to an entity *DynamicField* that is related to the *Analysis* entity via the relation *HasDynamicField* as depicted in Fig. 5.

As our dashboard is focused on the analytical results, we then further refined our graph data model at this place. There were multiple ways of how to restructure the model around the specific replacement of the *DynamicField* placeholder depending on the value type of the analytical result:

1. A dynamic field can be restructured when pulling the respective entity node into the specific analysis by setting the stored name or key of the field as new attribute name. Such a restructuring is suitable if there are dedicated fields with a huge or even infinite active domain that are also independent from other fields (e.g., a field with name *q* for some measured numerical value would be a reasonable extension to the corresponding analysis entity node as additional attribute). Creating an individual node for each possible value, in contrast, is obviously not feasible if there are too many possibilities.
2. If the data type of the dynamic field is multi-valued (e.g., a list of identified mutations) or multiple dynamic fields constitute one logical entity that reasonably should be grouped together (e.g., some combination of fields represent an estimated result), a likelihood for the estimation and the next most likely estimation, the placeholder entity can be replaced with a more specific entity or set of entities. In our use case, one new node label named *Fusion* was inserted into the database that stands for a certain mutation or group of mutations. The information about each analysis that detected a fusion matching one of the mutations was then stored by establishing the relation *HasFusion* between the specific analysis node and the corresponding fusion node. This was an improvement as it resolved the dynamic fields even over different types of analysis identifying mutations. Additionally, this facilitated queries and the graph structure as patients with the same mutation(s) related to the same fusion node(s).

## Results

### Architecture and ETL

**Fig. 6:**
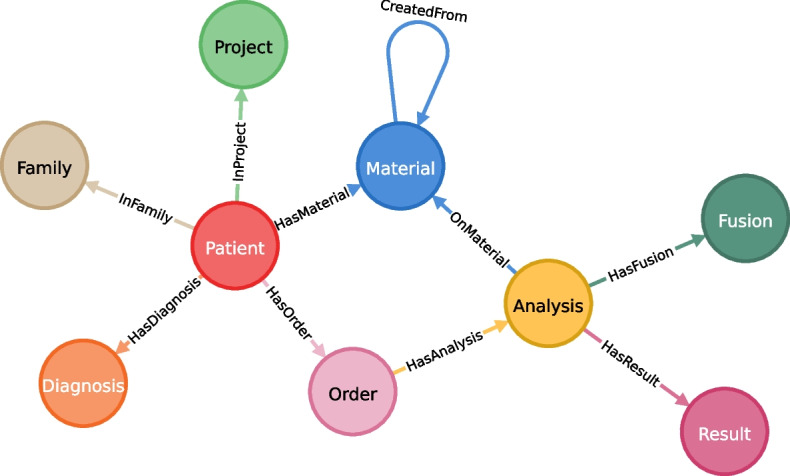
Graph database model. The final graph database model as applied in the Neo4j database behind the web-based dashboard in Graph4Med. The nodes of this graph represent the different domains of the objects stored in the database and the edges indicate the relationships between two objects

The final graph database model as obtained from the transformations of $\textbf{RG}$ (cf. Implementation) and implemented in the Neo4j graph database as backbone of our Graph4Med system is shown in Fig. 6. The nodes model the different domains (i.e., the classes of objects stored in the graph) and the edges model the relationships between objects. The implemented model was equal to the union $\mathbf {RG_1} \cup \mathbf {RG_2}$ after the applied transformations (right graphs from Figs. 3 and 5). The *Analysis* node was still a generic node label from which other labels for more specific analysis types (e.g., the label *ArrayCGHAnalysis* for Array-CGH analyses) were derived. For the sake of a less overloaded graph these are not shown in Fig. 6. Here, we also show the *Fusion* node as a restructuring of a dynamic field that was detected for different types of analysis (e.g., RNA sequence or Array-CGH analysis).

We used Python scripts to extract the patient cohort of pediatric ALL cases from the relational MS-SQL database server in an incremental fashion via SQL queries. Then, the obtained data were transformed into the graph data model using Neomodel [21], an Object Graph Mapper (OGM) for the Neo4j database system, and loaded into the Neo4j database instance. First the patients’ personal information (i.e. id, name, age, gender) were queried and added as nodes to mark the cohort. Subsequently all other related data (e.g., projects, orders, analyses) were queried for each patient of the cohort and linked to the related nodes according to the schema. We focused on the retrieval of general information about the patient and related entities, e.g., diagnoses or lab results. The analytics-related data concentrated on the results of various assays and methods, instead of raw data, e.g., raw NGS data, raw karyotype data. Our system is not intended to store the raw data, but instead the analytical results and related information, e.g., the materials on which the specific analyses were carried out, that contribute to the visualization regarding the ALL cases in our proposed visualization tool. The setup of the Neo4j database took approx. 50-55 minutes and was scheduled to be updated once every night.

### NeoDash dashboard builder

Based on this Neo4j database, the web-based dashboard building tool NeoDash [18] was deployed in a web server next to Neo4j and used to build a dashboard for the visualization and analysis of the ALL cohort. Once the NeoDash web application is setup, it can be connected to any Neo4j database instance for a convenient development and usage of dashboards for displaying and analyzing the stored data. The main components of a dashboard are one or more pages that each contain a collection of reports, i.e., some sort of visualization of information. The reports are populated with the results of Cypher queries that are executed via the dashboard against database on-the-fly. A built-in query editor enables to formulate these queries for the reports based on the selected chart type, e.g., a bar chart. The definition of the dashboard structure itself can also be stored in Neo4j for a convenient versioning, sharing and on-demand loading of the developed dashboards. The modularity of dashboard pages with multiple reports increases the flexibility regarding the extension of the dashboard by adding reports or new pages on demand.

Our analysis dashboard consists of several pages to grant different views on the data being investigated. Each page comprises multiple reports presenting tabular, graph-based, or chart-based visualizations of the patient data. In particular, our dashboard incorporates pages for the general overview and statistics of the full cohort, the detailed analysis of a specific subgroup of patients and the analysis of a single patient including the similarity comparison to other patients.

### Cohort view

**Fig. 7:**
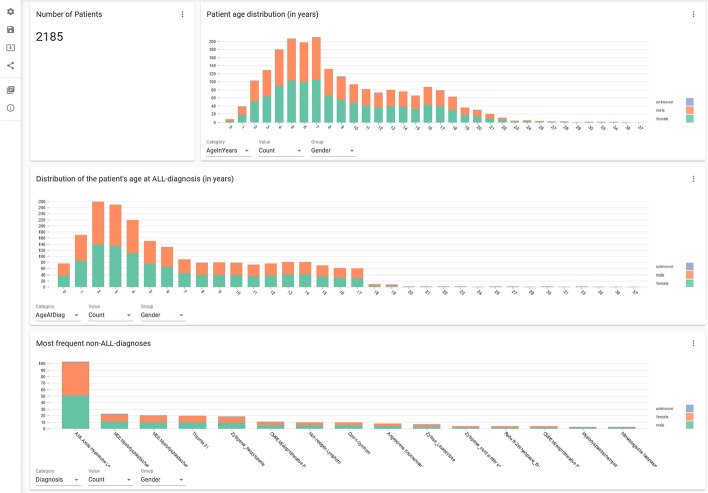
Cohort view. Dashboard page for general cohort information and statistics such as gender and age distribution. The three drop down fields below each bar plot allow to choose distributions other than gender and age. Note: due to data privacy reasons, the plots shown here were made from artificially generated data

In the previously used relational database of this pediatric ALL cohort, it was always cumbersome to retrieve overall statistics. Doctors and researchers were required to perform the counting and selections from the exported tables themselves, which is time consuming and error-prone. It was almost impossible for them to generate visualizations of cohort statistics. In contrast, with our tool Graph4Med, they can now easily obtain up-to-date statistics and visualizations immediately. For example, in the cohort view page of our dashboard, the top two bar plots show the distribution of current age (top) or age at diagnosis (middle) (Fig. 7), which are both additionally grouped by the gender of the cohort patients. (Note: Due to data privacy reasons, we do not show the plots from the real data, but instead from artificially generated data). The number to the top left indicates the size of the cohort. In these bar plots, the stacked colors indicate the distribution grouped by gender. Green and orange indicate female and male, respectively. The bottom bar plot shows various non-ALL diagnosis grouped by gender. Because our cohort had a small percentage of non-ALL cases such as Acute Myeloid Leukemia (AML), pediatric and non-pediatric Myelodysplastic Syndrome (MDS), or Trisomy 21, it is very helpful to have the number and gender distribution of these comorbidity cases. These plots are useful for identifying relationships between age/gender and disease. Moreover, our dashboard can displays other useful information in addition to age/gender: The three drop down fields below the bar plot allow users to choose the values to plot on the X-axis, Y-axis and color group. For example, it is possible to show frequencies instead of patients counts. Similarly, it is possible to display distributions of MRD (minimal residual disease) or material type instead of gender.

### Subgroup view

**Fig. 8:**
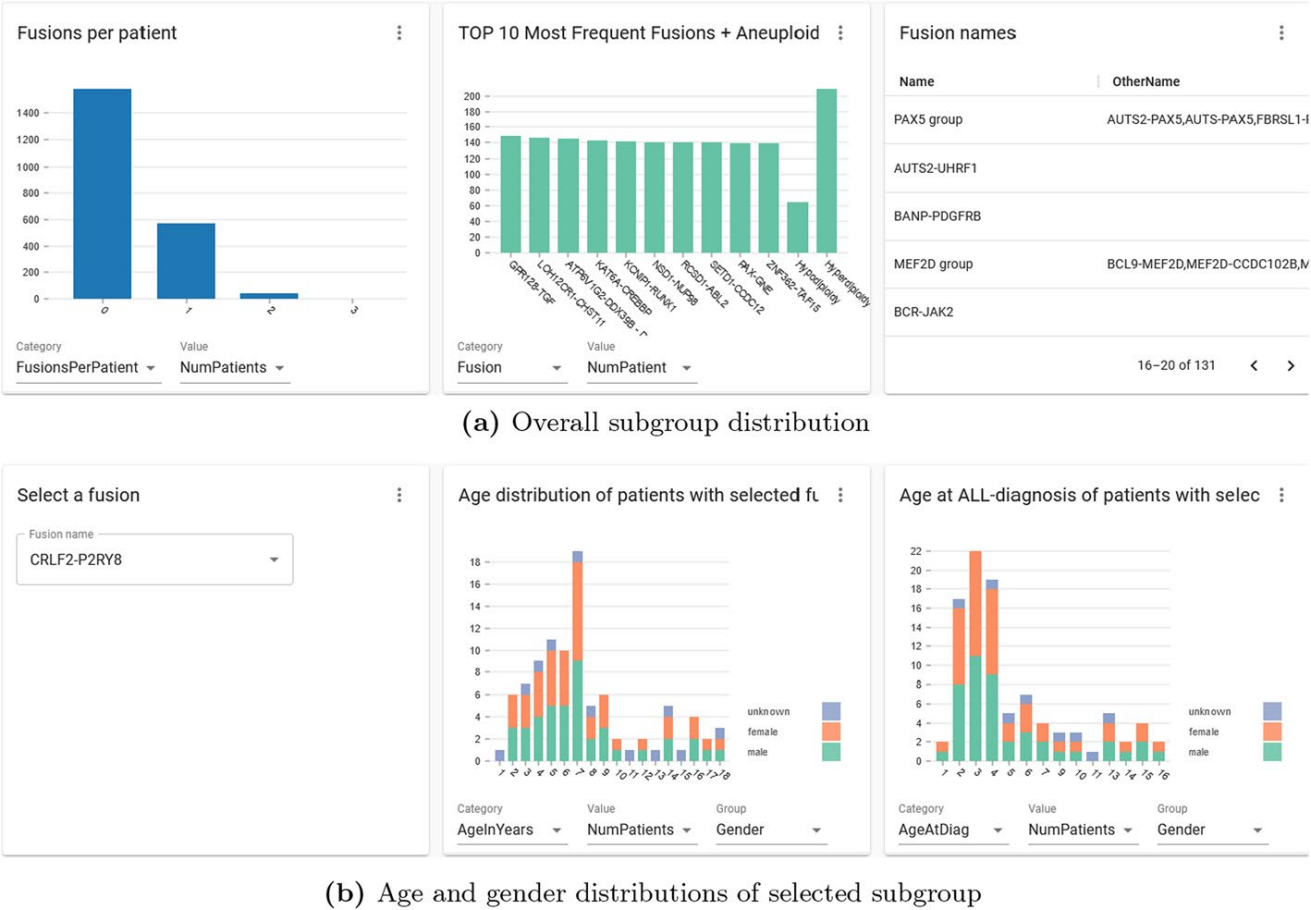
Subgroup distribution. **a** The left panel shows the number of fusions per patient. The middle panel shows the distribution of 10 most frequent B-ALL subgroups. The table on the right lists all the fusion names and their aliases. **b** Age and gender distribution for selected fusion, “CRLF2-P2RY8” in this case. *Note*: Due to data privacy reasons, the plots shown here do not reflect the real distributions

As mentioned previously, leukemia is mostly driven by gene fusions and aberrant chromosome numbers. They are the main factors for deciding B-ALL subgroups and play important roles in risk stratification [17]. In Fig. 8a, we demonstrated the number of fusions per patient in the left panel, and a distribution of major B-ALL subgroups in the middle panel. The table on the right shows all the names of fusions and their aliases used in the database.

Because gene fusion and aneuploidy information are important for leukemia, we implemented a function to select certain fusion or aneuploidy type, and then visualize patients’ distribution within this subgroup. In the left panel of Fig. 8b, there is an auto-completing selection field where users can enter the name of a subgroup, for example, CRLF2-P2RY8 was chosen in this case. In the middle and right panel of Fig. 8b , we display the age and gender distributions for this selected subgroup.

**Fig. 9:**
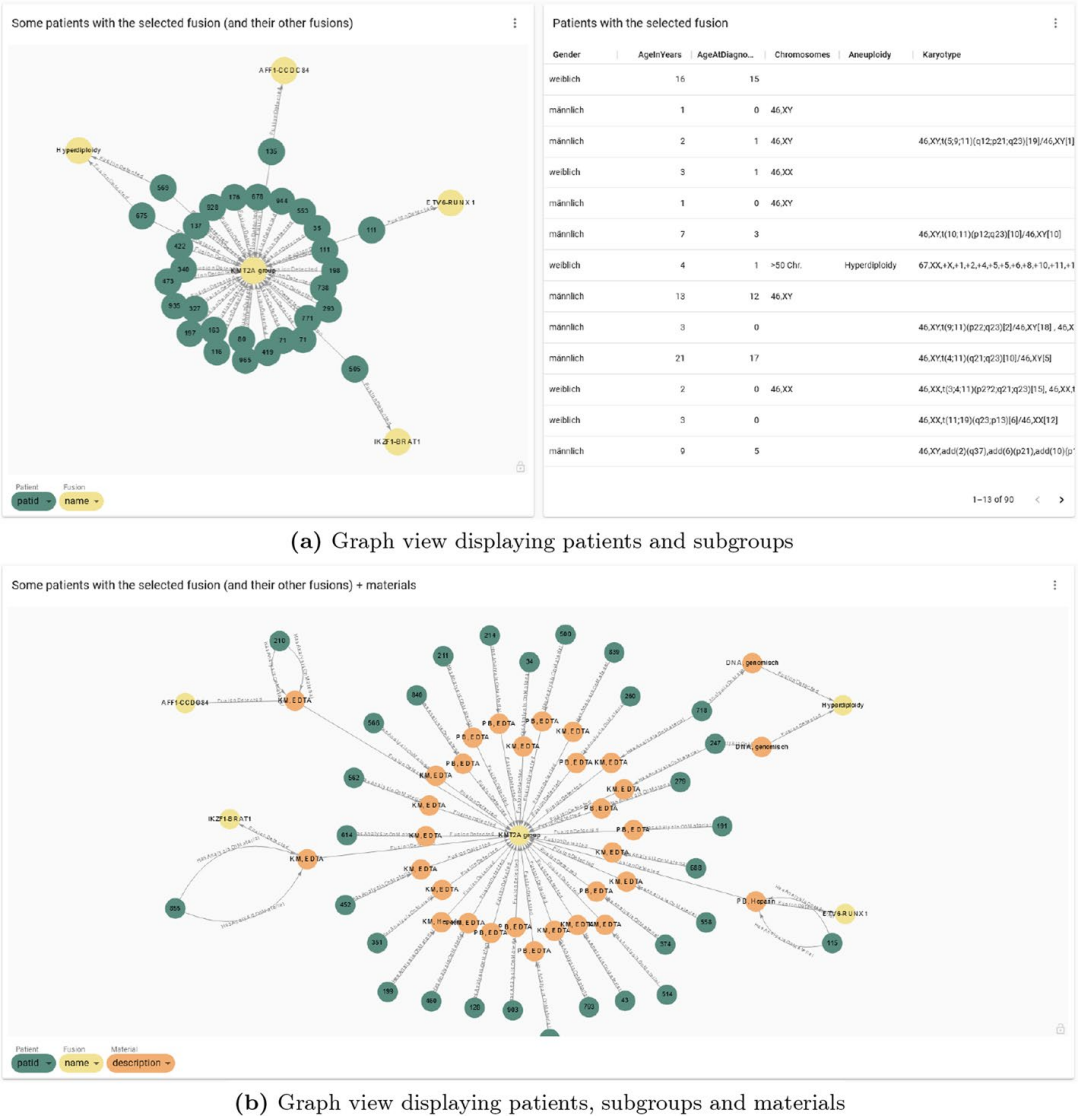
Graph view of relationships. **a** Graph view of a selected subgroup. It shows the relationship between patients and subgroups. The table on the right lists all the patients belonging to this subgroup with their additional information such as age, karyotype, chromosomes etc. **b** Different features of patients (color of node) can be integrated, for example, patients materials (orange nodes) were chosen here as additional information. *Note*: due to data privacy reasons, the plots and table shown here were made from artificially generated data

We also developed a graph view to demonstrate the relationships among patients. After selecting a certain subgroup, all the patients of this subgroup are displayed in a table (right panel of Fig. 9a). A maneuverable graph showing the relationships between patients (green nodes) and subgroups (yellow nodes) are displayed on the left. This graph can show different nodes depending on the needs. For example, in Fig. 9b, material information (orange nodes) were added in relation to the patients and subgroup. Similarly any other features (color of nodes) mentioned in Fig. 6 can be added. The exact value to display inside each node can be chosen by the drop-down menu on the bottom, which has the same color scheme as nodes (more explanation in next section and Table 1). Our graph is flexible in that it can not only display any feature (color of nodes) of interests but users may also (1) drag the nodes for easier visualizations; (2) hover over or click on nodes and relationships to inspect their properties; (3) zoom in/out on any region of the graph.

**Table 1:** List of variables to display at graph reports for some node labels

Node label	Alternative attribute
Patient (dark blue)	id, name, gender, date of birth
Diagnosis (pink)	name, icd code
Material (brown)	id, type, description
Order (orange)	id, type, date
Analysis (gray)	id, analytical result
Fusion (light blue)	name, other names

### Individual patient view

**Fig. 10:**
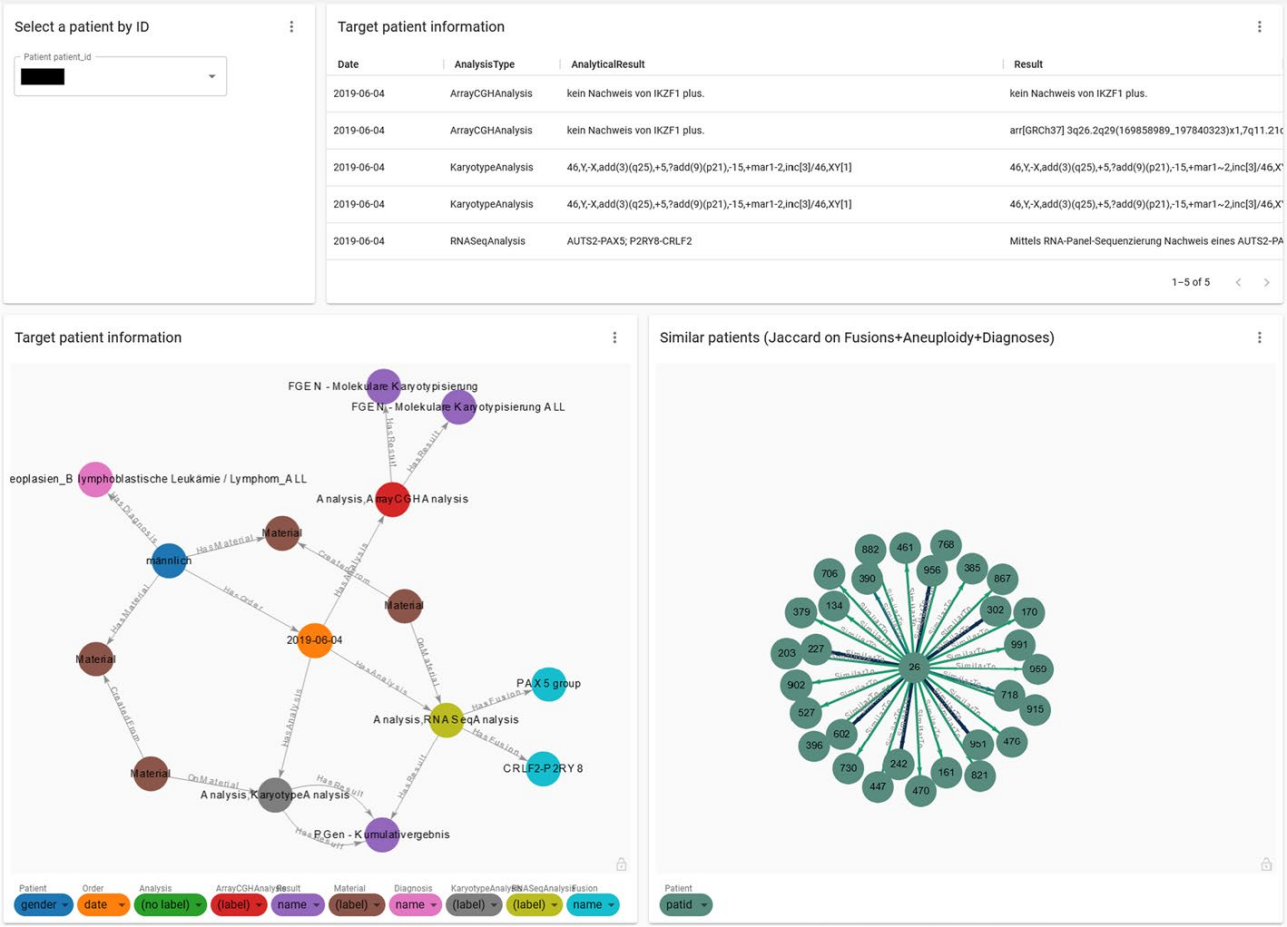
Individual patient view. Presentation of an individual patient’s information in a table and graph (top right and bottom left panel). Bottom right is the result of the similarity search across the entire cohort with the selected patient in the middle. Note: due to privacy reasons, the plots shown here were made from artificially generated data

The traditional relational database was designed based on the management and health care for an individual patient. Its functionality is sufficient to retrieve information of a particular patient (e.g., a doctor checks the lab results and plans the treatment for one patient). Here, we enhance this functionality by displaying an individual’s information in a compact, maneuverable graph. By a single glance, the users can grasp most of the details instead of going through the cumbersome and error-prone tabular process. The bottom left of Fig. 10 shows such a graph where different colors of nodes represent different types of information (features) for the selected patient. The user can dynamically adapt the value displayed on the nodes through the small drop-down menus at the bottom of the graph. In this way, the node text can be set to any of the node attributes or some basic property such as the node label according to the data schema. For instance, currently in Fig. 10 the dark blue node presents the patient’s gender; the brown nodes depict a single material linked to the patient and analysis; the light grey nodes represent the fusions identified, which are PAX5 and CRLF2-P2RY8 in this case. Table 1 gives an overview for the alternative values that could be displayed on the nodes of each type (color). Furthermore, the tabular report in Fig. 10 gives an overview over the subgroup with one row per patient. The columns can be sorted and filtered and the user can also decide which columns to display or hide (e.g., the column with the patient ID is hidden here).

Graph4Med can also generate a patient similarity graph, which is shown on the bottom right of Fig. 10. Here, we implemented a very simple similarity algorithm based on diagnosis, gene fusions, and aneuploidy which can be treated as a fusion. Depending on the needs of the specific medical use case, a more sophisticated similarity measure could be developed in the future. In our use case, the Jaccard index $J(A, B) = \frac{A \cap B}{A \cup B}$ was applied to the target patient and all other candidates. It yields a value between 0 and 1 where 1 indicates maximum similarity between target and candidate patient and 0 indicates no similarity between them. In the example shown on the bottom right of Fig. 10, the target patient (the node in the middle) is connected to all the patients that have a similarity exceeding a certain threshold. The width and color of the edges between nodes scale with the level of similarity: Bolder and darker connections represent a strong similarity and vice versa. In this example, we see two levels of similarity, thin light-green and thick blue.

## Discussion

In Graph4Med, we converted a relational database to a graph database in Neo4j and further built a dashboard on it. This tool is very well liked by our end users—clinicians, researchers and lab scientists. It not only provides meticulous visualizations other than tables and statistics of the whole cohort, but also enables to search patient subgroups based on fusions and aneuploidy, which are the most important factors in stratifying leukemia patients. The former system did not support the search for patient subgroups nor the computation of statistics whereas these important aspects were effortlessly included in the dashboard reports. We also implemented a small algorithm to display patient similarities. The success of Graph4Med in the pediatric ALL database has already prompted interests among clinicians from other disease areas.

Table 2 summarizes the implementation of key features for the old and new system that have driven the development of Graph4Med. Our dashboard is designed to be user-friendly with flexibility and interactivity to improve the efficiency of the current clinical practice. To this end, it overcomes several limitations of the former system, e.g., by providing various visualizations, broad overviews and summarizing statistics. The key feature to navigate individual cases is still kept while Graph4Med also enables the detection of complex relationships among patients and subpopulations in the dashboard pages. It allows each user to individually modify the visualizations and adapt them to his/her specific needs. (1) Users can select the contents to be displayed in the nodes on-demand. (2) Because there are very complex data structures in the original database even for one patient (see Fig. 9b), it is impossible to display all the nodes when we have more than 5 patients. Our tool made it possible to choose what type (color) of nodes to display. (3) The Cypher queries for plots and tables are embedded in the dashboard and the query language is the only prerequisite for adding additional reports. It is possible for non-IT users to modify parameters and obtain the plots they desire.

**Table 2:** Comparison between the old relational system (RDB) and Graph4Med with evaluations from our end users. The figure references relate to implementation examples

Feature	RDB	Graph4Med	User comments
Overview & general cohort statistics	✗	✓ (Fig. 7)	In the RDB, the DB administrator queries for the desired information summarizing a patient cohort in a table from which plots are generated manually with third-party tools. This usually takes several weeks in our practice, especially, if the query has to be adapted after seeing the first version of the plot.
Visualization of cohort/subpopulation	✗	✓ (Fig. 8)	
Visualization of relationships among patients	✗	✓ (Fig. 9a)	
Navigate through individual cases	✓	✓	
Gather a patient profile & visualize various data of individual cases	✗	✓ (Figs. 9b and 10)	In the old system, users have to browse multiple tables to gather a profile of an individual case and there is no possibility of visualizing relationships among various data in such a profile (cf. Fig. 10)
Eliminate redundant answers	✗	✓	
Aggregate results	✗	✓	
Fusion-based search	✗	✓ (Figs. 8b and 9a)	Gene fusions are the most important drivers for leukemia, and also the main bases for patient stratification, thus, searching for patients with the same fusion is very useful.
Patient similarity search	✗	✓ (Fig. 10)	

As the structure of the dashboard itself is stored in the Neo4j database, it can be updated and extended easily. The users are allowed to share and load different versions of the dashboard at any time. Furthermore, this even gives the option to have access to multiple independent dashboards focusing on different use cases or research questions on the same underlying data set. The graph database also implicitly removes redundant answers with the graph structure and data aggregation becomes feasible than with the complex SQL queries and overloaded tables. This results in less overhead for the users who can skip the time intense step of filtering the redundant information from the tables.

Medical databases are constantly updated. Therefore, we update the Graph4Med database every night to keep the dashboard up-tp-date. Graph4Med currently has 2185 patients with 4,919 analyses and 723 fusions detected and currently contains 64,677 nodes and 77,129 relationships with a total size of approx. 400 MB. It takes about 50–55 min to update the whole database.

### Comparison with related medical dashboards

In Table 3 we compare Graph4Med with other medical visualization systems or dashboards on several high-level features. The column “System” lists the different medical visualization systems and dashboards. For each system, the table states the actual database management sytem (col. “DBMS”) as well as the offered types of interfaces (col. “Interface”), e.g., a web application or command line interface (CLI). We evaluated the interactivity, i.e., the possibility to interact with the plots of the dashboard, and the flexibility, i.e., the option to effortlessly extend the dashboard with new visualizations or reports, of the dashboards and the results are shown in the columns “Int” and “Flex”, respectively. The table column “Coll” refers to the capability of the system for collaboration in the sense of versioning or sharing extended dashboard versions with other users and “Exp” to the ability to export charts or download data from the tool. Table 3 also contains a column “Data” that summarizes the types of data dealt with in the corresponding system.

**Table 3:** Comparison of medical visualization systems and dashboards. The implementation details on the database (“DBMS”), available interfaces (“Interface”) and the data (“Data”) are listed. The features interactivity (“Int”), flexibility (“Flex”), collaboration (“Coll”) and export(“Exp”) are assessed qualitatively

System	DBMS	Interface	Int	Flex	Coll	Exp	Data
MyPal [22]	Unknown	Web, Mobile App	✓	✗	✗	✓	Patient data, demographics, treatment, patient-reported outcomes
CovidGraph [23]	Neo4j	Web	✓	✗	✗	✓	Publications, patents, clinical trials, genes, transcripts, proteins, pathways, statistics, systems biology data
OncoKB [24]	MySQL	Web, CLI	✓	✗	✗	✓	Cancer mutations, drugs with corresponding cancer type & targeted mutations
BioGraph [16]	Neo4j	Web	✓	✗	✗	✓	Genes, proteins, miRNA-related data, pathways, functional annotations
LinkedImm [12]	Neo4j	Web	✓	✗	✗	✓	Immunological data, genes, pathways, transcriptional profiling data
Graph4Med	Neo4j	Web	✓	✓	✓	✗	Patient diagnostic, genomic analysis results (incl. karyotyping, RNASeq, arrayCGH)

### Limitations

During our implementation, we also observed some technical limitations of building the dashboard by NeoDash and Neo4j. For example, (1) One cannot export the report charts, graphs or tables directly from the dashboard; (2) One can not select a certain sub-population after choosing a fusion type. It would be desirable if users can choose which patients from the table to include in the graph report (Fig. 9a). We would also like to mention that due to the complexity of our previously used database, we did not convert all the different analysis types to our graph database. However, the missing analysis or any novel techniques can be integrated seamlessly in accordance to previously incorporated analysis. The results of FISH analyses that are also employed to identify gene fusions are not incorporated yet, for instance, which leads to smaller numbers of ETV6-RUNX1 and BCR-ABL cases in the cohort statistics than they actually are.

As Neo4j and NeoDash are constantly improving, we should be able to improve the functionality of our dashboard such as exporting reports in the future. We are also interested to expand Graph4Med to other disease databases or to integrate additional sources of information. One future direction to extend Graph4Med would be to integrate more data for the diagnostic pipeline such as gene expression and point mutation results. Incorporating these results or additional resources with bioinformatics data into Graph4Med could further facilitate the analytical capabilities of the system. We could also have more complex algorithms for measuring patient similarity when the use case is bigger and contains a rich diversity of diseases. For example, our collaborator at the Department of Human Genetics of the Medizininsche Hochschule Hannover also has a database for various, rare genetic diseases. We could design a new similarity algorithm considering various factors including age, gender, symptom, genetic factors. In the case of rare diseases, suggestions from patient similarity search will be especially useful in pinpointing treatment options.

## Conclusion

In our work, we developed a flexible medical visualization tool including a web-based dashboard on top of a Neo4j graph database storing the application data. We presented a method on how to convert a relational database schema to a graph data model for the easy implementation of our dashboards with Cypher queries against the stored data graph. The visualizations provide the analytical capabilities in a convenient and interactive fashion that were not possible in the old system. Our work proves the flexibility and feasibility of a graph database for managing medical data as it allows for an intuitive representation of the structure of the medical data schema.

## Data Availability

The clinical data used in our approach are not shared due to the preservation of the patients’ privacy. A public demonstrator with artificial data can be found on the Graph4Med website. Source code is located in the project repository.

## Abbreviations

AML: Acute myeloid Leukemia
Array-CGH: Array-based Comparative Genomic Hybridization
ALL: Acute lymphoblastic leukemia
CLI: Command line interface
DBMS: Database management system
DNA: Deoxyribonucleic acid
FISH: Fluorescence in situ hybridization
FK: Foreign key
MDS: Myelodys-plastic syndrome
microRNA: Micro ribonucleic acid
MRD: Minimal residual disease
NGS: Next generation sequencing
OGM: Object graph mapper
PK: Primary key
SQL: Structured query language
